# Supplementary material for: P-TRAP: a Panicle Trait Phenotyping tool
Source: BMC Plant Biol. 2013 Aug 29;13:122. doi: 10.1186/1471-2229-13-122 (PMC3848748; doi:10.1186/1471-2229-13-122)
Supplement: Additional file 7 — P-TRAP source code. The Netbeans project that contains the source code of the application. [file 1471-2229-13-122-S7.zip › javadoc.zip!/javadoc/org-bioalg-imageprocessor/org/bioalg/imageprocessor/ColoredImage.html]

ColoredImage (Image Processor)


JavaScript is disabled on your browser.


- Package
- Class
- Tree
- Deprecated
- Index
- Help

*org.bioalg.imageprocessor 1.0*

- Prev Class
- Next Class

- Frames
- No Frames

- All Classes

- Summary:
- Nested |
- Field |
- Constr |
- Method

- Detail:
- Field |
- Constr |
- Method


org.bioalg.imageprocessor

## Class ColoredImage

- java.lang.Object
- - org.bioalg.imageprocessor.ColoredImage

- ---

    

  ```
  public class ColoredImage
  extends java.lang.Object
  ```

  The colored image class.

  Author:
  :   faroqal-tam

- - ### Constructor Summary

    Constructors

    Constructor and Description

    `ColoredImage(java.awt.image.BufferedImage bufferedImage)`

    Create a colored image from a bufferedImage.

    `ColoredImage(int height,
    int width)`

    An empty image constructor.
  - ### Method Summary

    Methods

    | Modifier and Type | Method and Description |
    | --- | --- |
    | `int` | `getHeight()` |
    | `RGBPixel[][]` | `getMatrix()` |
    | `RGBPixel` | `getRC(int r, int c, RGBPixel border)` Get the pixel at a given row and column. |
    | `int` | `getWidth()` |
    | `void` | `setMatrix(RGBPixel[][] matrix)` |
    | `void` | `setRC(int r, int c, RGBPixel val)` Sets the pixel at the row r and column c by another pixel. |
    | `AImage` | `simpleSegmentation(HSBPixel objectBPixel, HSBPixel backgroundBPixel)` Performs a simple color thresholding by classifying the pixels based on the distance to the background and object the as background (0) or object (255) by measuring |
    | `AImage` | `simpleSegmentation(RGBPixel objectBPixel, RGBPixel backgroundBPixel)` Performs a simple color thresholding by classifying the pixels based on the distance to the background and object the as background (0) or object (255) by measuring |

    - ### Methods inherited from class java.lang.Object

      `clone, equals, finalize, getClass, hashCode, notify, notifyAll, toString, wait, wait, wait`

- - ### Constructor Detail


    - #### ColoredImage

      ```
      public ColoredImage(int height,
                  int width)
      ```

      An empty image constructor.

      Parameters:
      :   `height` -
      :   `width` -


    - #### ColoredImage

      ```
      public ColoredImage(java.awt.image.BufferedImage bufferedImage)
      ```

      Create a colored image from a bufferedImage.

      Parameters:
      :   `bufferedImage` - a `BufferedImage` object.
  - ### Method Detail


    - #### getRC

      ```
      public RGBPixel getRC(int r,
                   int c,
                   RGBPixel border)
      ```

      Get the pixel at a given row and column.

      Parameters:
      :   `r` - the row
      :   `c` - the column
      :   `border` - if =-1 it will uses the border pixel if the r or c out of the image domain.

      Returns:
      :   an `RGBPixel` object.


    - #### setRC

      ```
      public void setRC(int r,
               int c,
               RGBPixel val)
      ```

      Sets the pixel at the row r and column c by another pixel.

      Parameters:
      :   `r` - the row
      :   `c` - the column
      :   `val` - the new pixel value.


    - #### getHeight

      ```
      public int getHeight()
      ```


    - #### getWidth

      ```
      public int getWidth()
      ```


    - #### simpleSegmentation

      ```
      public AImage simpleSegmentation(RGBPixel objectBPixel,
                              RGBPixel backgroundBPixel)
      ```

      Performs a simple color thresholding by classifying the pixels based on
      the distance to the background and object the as background (0) or object
      (255) by measuring

      Parameters:
      :   `objectBPixel` -
      :   `backgroundBPixel` -

      Returns:


    - #### simpleSegmentation

      ```
      public AImage simpleSegmentation(HSBPixel objectBPixel,
                              HSBPixel backgroundBPixel)
      ```

      Performs a simple color thresholding by classifying the pixels based on
      the distance to the background and object the as background (0) or object
      (255) by measuring

      Parameters:
      :   `objectBPixel` -
      :   `backgroundBPixel` -

      Returns:
      :   an `AImage` object


    - #### getMatrix

      ```
      public RGBPixel[][] getMatrix()
      ```


    - #### setMatrix

      ```
      public void setMatrix(RGBPixel[][] matrix)
      ```


- Package
- Class
- Tree
- Deprecated
- Index
- Help

*org.bioalg.imageprocessor 1.0*

- Prev Class
- Next Class

- Frames
- No Frames

- All Classes

- Summary:
- Nested |
- Field |
- Constr |
- Method

- Detail:
- Field |
- Constr |
- Method
